# Genomic tumor evolution dictates human medulloblastoma progression

**DOI:** 10.1093/noajnl/vdae172

**Published:** 2024-10-05

**Authors:** Yana Ruchiy, Ioanna Tsea, Efthalia Preka, Bronte Manouk Verhoeven, Thale Kristin Olsen, Shenglin Mei, Indranil Sinha, Klas Blomgren, Lena-Maria Carlson, Cecilia Dyberg, John Inge Johnsen, Ninib Baryawno

**Affiliations:** Childhood Cancer Research Unit, Department of Women’s and Children’s Health, Karolinska Institutet, Stockholm, Sweden; Childhood Cancer Research Unit, Department of Women’s and Children’s Health, Karolinska Institutet, Stockholm, Sweden; Childhood Cancer Research Unit, Department of Women’s and Children’s Health, Karolinska Institutet, Stockholm, Sweden; Childhood Cancer Research Unit, Department of Women’s and Children’s Health, Karolinska Institutet, Stockholm, Sweden; Childhood Cancer Research Unit, Department of Women’s and Children’s Health, Karolinska Institutet, Stockholm, Sweden; Department of Immunology, Genetics, and Pathology, Uppsala University, Uppsala, Sweden; Center for Regenerative Medicine, Massachusetts General Hospital, Boston, Massachusetts, USA; Childhood Cancer Research Unit, Department of Women’s and Children’s Health, Karolinska Institutet, Stockholm, Sweden; Department of Molecular Biosciences, The Wenner-Gren Institute, Stockholm University, Stockholm, Sweden; Paediatric Oncology, Karolinska University Hospital, Stockholm, Sweden; Childhood Cancer Research Unit, Department of Women’s and Children’s Health, Karolinska Institutet, Stockholm, Sweden; Paediatric Oncology, Karolinska University Hospital, Stockholm, Sweden; Childhood Cancer Research Unit, Department of Women’s and Children’s Health, Karolinska Institutet, Stockholm, Sweden; Childhood Cancer Research Unit, Department of Women’s and Children’s Health, Karolinska Institutet, Stockholm, Sweden; Childhood Cancer Research Unit, Department of Women’s and Children’s Health, Karolinska Institutet, Stockholm, Sweden; Childhood Cancer Research Unit, Department of Women’s and Children’s Health, Karolinska Institutet, Stockholm, Sweden

**Keywords:** medulloblastoma, single-cell sequencing, tumor heterogeneity

## Abstract

**Background:**

Medulloblastoma (MB) is the most common high-grade pediatric brain tumor, comprised of 4 main molecular subgroups—sonic-hedgehog (SHH), Wnt, Group 3, and Group 4. Group 3 and Group 4 tumors are the least characterized MB subgroups, despite Group 3 having the worst prognosis (~50% survival rate), and Group 4 being the most prevalent. Such poor characterization can be attributed to high levels of inter- and intratumoral heterogeneity, making it difficult to identify common therapeutic targets.

**Methods:**

In this study, we generated single-cell sequencing data from 14 MB patients spanning all subgroups that we complemented with publicly available single-cell data from Group 3 patients. We used a ligand–receptor analysis tool (CellChat), expression- and allele-based copy-number variation (CNV) detection methods, and RNA velocity analysis to characterize tumor cell–cell interactions, established a connection between CNVs and temporal tumor progression, and unraveled tumor evolution.

**Results:**

We show that MB tumor cells follow a temporal trajectory from those with low CNV levels to those with high CNV levels, allowing us to identify early and late markers for SHH, Group 3, and Group 4 MBs. Our study also identifies *SOX4* upregulation as a major event in later tumor clones for Group 3 and Group 4 MBs, suggesting it as a potential therapeutic target for both subgroups.

**Conclusion:**

Taken together, our findings highlight MB’s inherent tumor heterogeneity and offer promising insights into potential drivers of MB tumor evolution particularly in Group 3 and Group 4 MBs.

**Key Points**

- Medulloblastoma (MB) tumor cells follow a temporal trajectory from those with low copy-number variation (CNV) levels to those with high CNV levels representing “earlier” and “later” states.
- Identification of these states can be used to identify early and late markers in MB subgroups.
- *SOX4* is the only common marker upregulated in later clones in all Group 3 and Group 4 MB tumors presenting a potential therapeutic target.

**Importance of the Study**

We show that using computational methods, it is possible to identify early and late cell states in medulloblastoma tumors, which in turn allows the identification of state-specific markers. These markers can therefore represent potential therapeutic targets. Most notably, we identify *SOX4* as the only shared marker between later clones of every Group 3 and Group 4 tumor analyzed, offering a promising therapeutic target for these patients.

Medulloblastoma (MB) is the most common malignant pediatric brain tumor—comprises 4 main molecular subgroups (sonic-hedgehog [SHH], Wnt, Group 3, and Group 4), all characterized by different genetic, epigenetic, and genomic alterations, prognoses, and treatment approaches.^1^ Of these 4 subgroups, Group 3, and Group 4 MBs are the most poorly characterized, despite Group 3 having the worst prognosis of all subgroups,^2^ and Group 4 being the most common.^3^ The main challenges in characterizing these tumors lie within their intra-subgroup heterogeneity, and previous attempts at classifying them using single-cell omics into smaller subtypes^4^ have not yet translated into different risk stratifications or clinical treatments. Instead, they further underlined the high level of heterogeneity these tumors possess. Importantly, there is still a need to define the clonal architecture and evolutionary culprits in human MB.

To provide novel insights characterizing this intratumoral heterogeneity and identify potential therapeutic targets, we have performed single-cell RNA sequencing on 14 MB samples spanning all 4 subgroups. Due to the therapeutic challenges still encountered by SHH, Group 3, and Group 4 MBs, this study focused on providing novel insights into the tumor evolution of these subgroups through the incorporation of publicly available single-cell datasets.^5^ Our final dataset comprised 20 patient samples and a total of 83 405 single cells. First, we describe the cellular composition and the intercellular signaling patterns within these tumors. We then investigate genomic and genetic changes underlying the clustering of these cells and their communication by performing a copy-number variation (CNV) analysis. After deriving the cellular trajectories within each subgroup by using RNA velocity analysis^6^ and combining them with identified genomic changes to show the movement of trajectories from cells with low CNV levels to those with high CNV levels, we identify early and late tumor markers in all 3 subgroups. Finally, we detect individual clones within each tumor by employing a haplotype-aware somatic CNV analysis pipeline called *Numbat*.^7^ By performing a differential gene expression (DGE) analysis to identify markers of individual clones, we show that, remarkably, all Group 3 and Group 4 tumors demonstrate an upregulation of the *SOX4* gene in the later clones, which has been shown to play an oncogenic role in more than 20 different types of cancers.^8^

Our study further underlines the heterogeneity between and within the MB subgroups, pointing out the need for more personalized treatments. The identification of early and late tumor markers offers several promising gene candidates that could be targeted therapeutically. Finally, we propose *SOX4* as a potential therapeutic target for both Group 3 and Group 4 MBs.

## Materials and Methods

### Patient Material and Collection of Tumor Specimens

Medulloblastoma samples from 14 Swedish patients treated at Karolinska University Hospital were collected after parents or guardians had given written consent. All samples were collected according to permits approved by the Swedish Ethical Review Authority (2021-02481 and BTB 19001) and adhered to the Declaration of Helsinki. Clinical data were obtained from hospital records according to the ethical permit.

### Dissociation of Medulloblastoma into Single Cells

Single-cell suspensions of the tumors were obtained using the Brain Tumor Dissociation Kit (Miltenyi Biotec). After dissociation into single-cell suspension, samples were centrifuged at 1000 RPM for 5 min and resuspended in NeuroCult NS-A Basal Medium (Stem Cell Technologies) supplemented with 10% NeuroCult proliferation supplement (Stem Cell Technologies).

### Single-Cell Library Preparation and Sequencing

Medulloblastoma single-cell libraries were prepared using the Chromium single-cell 3’ reagent kit v3 (10x Genomics) according to the manufacturer’s recommendations. Libraries were sequenced on the NextSeq 500 platform (Illumina).

### Single-Cell Data Preprocessing and Identification of Cluster Markers

The Cell Ranger 6.0.2 pipeline was used to align the sequencing reads to the GRCh38-2020-A reference transcriptome^9^ and generate individual feature-barcode matrices. The generated matrices combined with matrices obtained from Riemondy et al.,^5^ (GEO accession number GSE155446) were then used for downstream processing using the Seurat pipeline (v. 4.3.0.1). Poor-quality cells were removed based on their mitochondrial counts (median + 2 standard deviations) and the gene counts threshold of 200. The additional doublets were identified using the R package DoubletFinder (v. 2.0.3),^10^ but were not excluded from the analysis since they represented a very small proportion of cells and did not form separate clusters. Cell cycle genes were identified using the CellCycleScoring function from the Seurat package to estimate the effect of the cell cycle on the clustering of cells and regressed out together with the mitochondrial gene counts. Stromal and immune clusters were identified in each patient’s dataset, and all other clusters termed “tumor” were then isolated and re-clustered. Individual patients’ tumor clusters were then integrated according to their MB subgroup by identifying common anchors using the *FindIntegrationAnchors* function using the top 3000 variable features for the downstream analysis. Principle component (PC) analysis identified the top 30 PCs that were used in the downstream *FindNeighbors* function, and cells were clustered with a resolution of 0.5. The clusters were then visualized using Uniform Manifold Projection and Approximation (UMAP). The *FindAllMarkers* function was then used to identify individual cluster markers based on *P*_adj_ value and pct.1/pct.2 ratio is necessary to label the clusters.

### Enriched Pathway Analysis

Enriched signaling pathways in each MB subgroup were identified using ClusterProfiler^11,12^ (v. 4.4.4) in R using a function *compareCluster* and the ReactomePA database.^13^ Only enriched pathways with a *P*_adj_ value of the enrichment score <.05 were selected followed by the selection of pathways with the highest GeneRatio scores representing the number of genes from the identified signaling pathways present in each subgroup.

### Ligand–Receptor Analysis

Cell–cell communication between the clusters was analyzed using CellChat (v. 1.6.1).^14^ Four types of cellular communication roles were identified: sender, receiver, mediator, and influencer. The sender and the receiver represented ligand-expressing and receptor-expressing cells, respectively. Cell clusters identified as mediators or influencers expressed genes that are involved in the signaling pathway or are co-stimulating it, respectively. None of the parameters needed to calculate the signal strengths were changed from the default.

### CNV Detection

CNVs were identified using the R package InferCNV (v. 1.12.0).^15^ Immune and stromal cells combined from all datasets were used as a reference. The gene order list supplied with the InferCNV package was used. A cutoff of 0.1 was used as a minimum average number of gene read counts among reference genes, as suggested in the InferCNV manual for the samples sequenced using 10x Genomics. A 6-state (i6) Hidden Markov Model was used to predict CNVs (the 6 states comprised complete loss (0x), loss of 1 copy (0.5x), neutral (1x), gain of 1 copy (1.5x), gain of 2 copies (2x), gain of more than 2 copies (3x)). The data obtained were integrated with the Seurat object of each MB subgroup using the *add_to_seurat* function with default parameters.

### Cell Trajectory Analysis

First, .bam files for each individual dataset were used to generate the .loom files containing spliced and unspliced RNA counts using the velocyto package (v. 0.17.16) in Python^6^ and Genome Reference Consortium Human Build 38 (GRCh38) was used as a reference genome.^9^ RNA velocity analysis was then performed to identify trajectories of individual cells within each subgroup using the scanpy (v. 1.9.3)^16^ and scvelo (v. 0.2.5)^17^ packages in Python. UMAP coordinates, count matrices including the metadata, and gene names for each dataset were extracted from the previously generated Seurat objects in R to create the anndata objects. Initial and terminal states were identified using the RNA velocity generalized dynamical model in combination with the CellRank package (v. 2.0.0) in Python.^18,19^

### Identification of Early and Late Markers

Using the input from the InferCNV analysis early and late markers were identified between low- and high-CNV clusters by assigning CNV status to each cluster based on the deviation from normal expression when compared to the reference cells. *FindAllMarkers* function from the Seurat package in R was then used to identify the differentially expressed genes (DEG). The identified genes were then filtered for positive log2FC values and those with the *P*-adjusted value lower than .05.

### Clonal Analysis

Different tumor clones in each individual tumor dataset were identified using the Numbat R package using expression, allele, and haplotype information.^7^ First, a pileup of single nucleotide polymorphisms (SNPs) was performed with cellsnp-lite^20^ using common SNP list in a variant call format from the 1000 Genome Project (hg38). Allele phasing was then performed using a phasing reference panel from the 1000 Genome Project (hg38). Finally, clones within each patient’s dataset were identified. Immune and stromal cells combined from all 20 datasets were used as a reference. Cell annotation file was provided to specify cell groups contained within the reference dataset, as well as for tumor cell annotation. Allele counts from the pileup and phasing step were used for the detection of CNVs in each clone. The transition probability used in the hidden Markov model (HMM) was kept at 1e−5 for every dataset irrespective of the complexity of the genomic landscape. Clonal markers were then identified using the DESeq2 package^21^ (v. 1.36.0) in R. The threshold for individual genes was set at >5 counts, and the genes with a *P*_adj_ value of <.1 were selected.

## Results

### Medulloblastoma Tumors Show High Levels of Intersubgroup Heterogeneity

**Figure 1.**
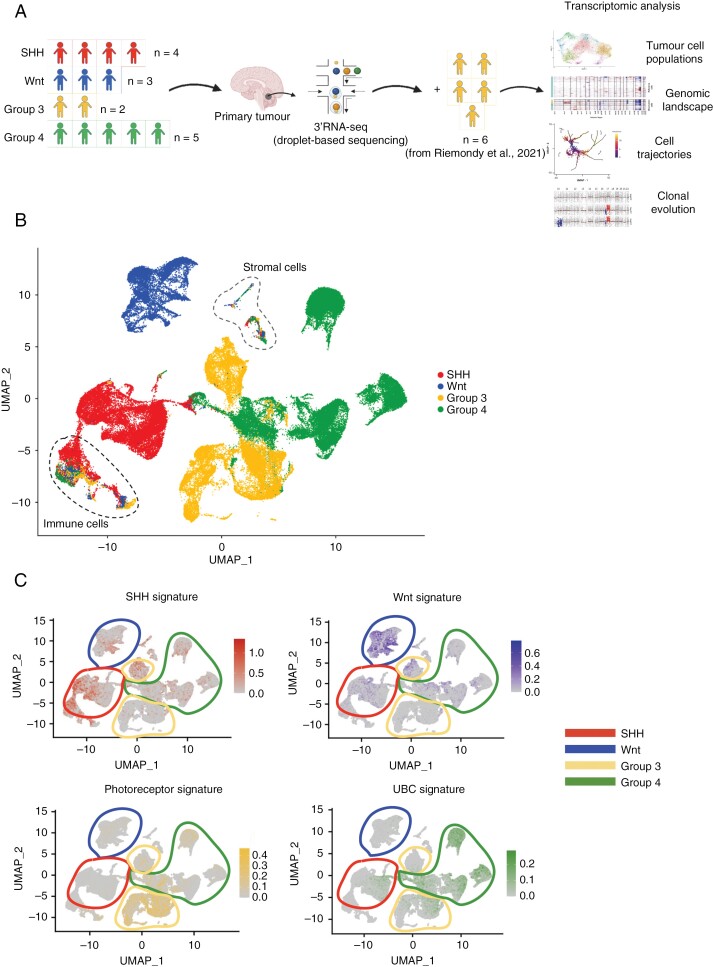
Molecular programs of human medulloblastoma (MB) tumors show high levels of subgroup specificity. (A) Primary tumors of 14 MB patients were collected at Karolinska University Hospital and contained all 4 MB subgroups further supplemented with 6 Group 3 samples from Riemondy et al. (2022) dataset.^5^ (B) UMAP projection of 83,405 single cells from 20 patients shows subgroup-specific clustering. (C) Previously published subgroup-specific signatures projected onto a UMAP of 83 405 single cells from our dataset.

**Figure 2.**
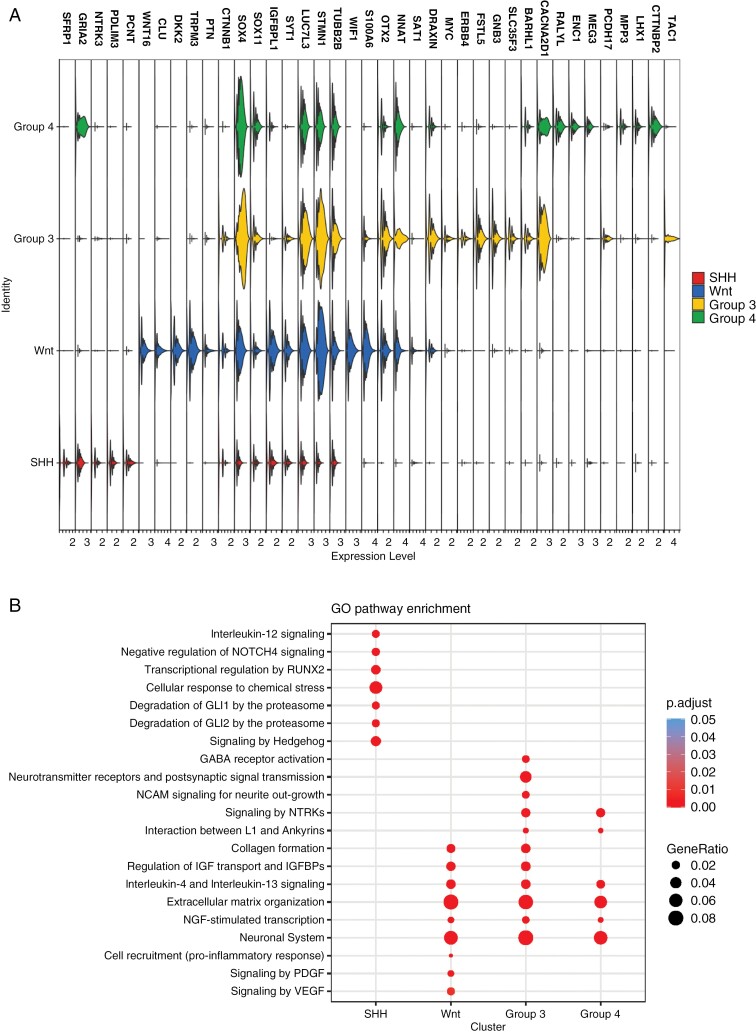
Medulloblastoma (MB) subgroups show upregulation of distinct gene groups and signaling pathways. (A) Violin plot showing selected subgroup-specific markers. (B) The enriched KEGG terms of top upregulated pathways in tumor cells for each MB subgroup. The statistical analysis was performed by over-representation test. The *P*-value was the number of randomized pairs exceeding the observed data. Benjamini–Hochberg method was used to adjust the *P*-value (<.05).

In order to investigate the genomic landscape of human MB tumors, we first obtained fresh surgical resections from 14 MB patients and performed single-cell RNA-seq profiling (10x Chromium) (Figure 1A). Each tumor sample was molecularly classified using DNA methylation arrays with the DKFZ classifier (Supplementary Table 1) and CNV profiles were inferred from the methylation data during the molecular classification (Supplementary Figure 1). Our dataset comprised all 4 molecular subgroups: 4 SHH samples (SHH-1—SHH-4), 3 samples from Wnt MB (Wnt-1—Wnt-3), 2 samples from Group 3 (Gr3-1, Gr3-2), and 5 samples from Group 4 MB (Gr4-1−Gr4-5) (Figure 1A). To complement our 2 Group 3 MB tumors, we further supplemented our dataset with 6 additional samples from a previously published single-cell dataset^5^ totaling 8 Group 3 samples (Figure 1A). After performing the quality control (see Materials and Methods and Supplementary Figure 2), we obtained the following numbers of single cells for each subgroup: SHH MB—20 688 cells, Wnt MB—11 535 cells, Group 3 MB—20 223 cells, Group 4 MB—30 979 cells (Figure 1B). We then projected the previously identified transcriptomic signatures,^22,23^ where we used >700 genes for Wnt MB, ~200 genes for SHH MB, ~200 genes for Group 3 MB, and >100 genes for Group 4 MB, onto our dataset (Figure 1C, Supplementary Table 2), which showed a strong overlap in both of the corresponding SHH and Wnt datasets, as well as photoreceptor and unipolar brush cell (UBC) signatures in our Group 3 and Group 4 datasets, respectively, consistent with their recently proposed cells of origin.^23,24^ In addition to these transcriptional programs, we also selected the top DEG that separate each MB subgroup within our dataset. More specifically, SHH tumors were characterized by *SFRP1* expression, a target of the SHH signaling, Wnt tumors showed expression of members of the Wnt signaling pathway including *CTNNB1*, encoding the main effector of the pathway beta-catenin, and *Wnt16*, while Group 3 and Group 4 tumors displayed expression of *FSTL5*, a known marker of non-Wnt/non-SHH MBs^25^ (Figure 2A). Additionally, Group 3 tumors showed upregulation of *MYC*—one of the most significant drivers of this MB subgroup^26^ (Figure 2A). Each subgroup was also characterized by the enrichment of distinct sets of signaling pathways (Figure 2B). The pathway enrichment analysis indicated upregulation of known pathways in tumor cells of SHH MB including Hedgehog, RUNX2, and interleukin-12 signaling as well as upregulation of proinflammatory cell recruitment, VEGF, and PDGF signaling in Wnt MB. Group 3 and Group 4 tumors showed an upregulation of neuronal pathways including “Transmission across chemical synapses” (Figure 2B).

Once we identified the activated gene expression programs and signaling pathways specific to each subgroup in our dataset, we performed an in-depth analysis of the tumor composition for each subgroup to better understand their genomic landscapes that underlie these transcriptional programs and signaling patterns and to identify potential druggable targets.

### Intratumoral Heterogeneity Reveals Distinctive Transcriptional Clusters

**Figure 3.**
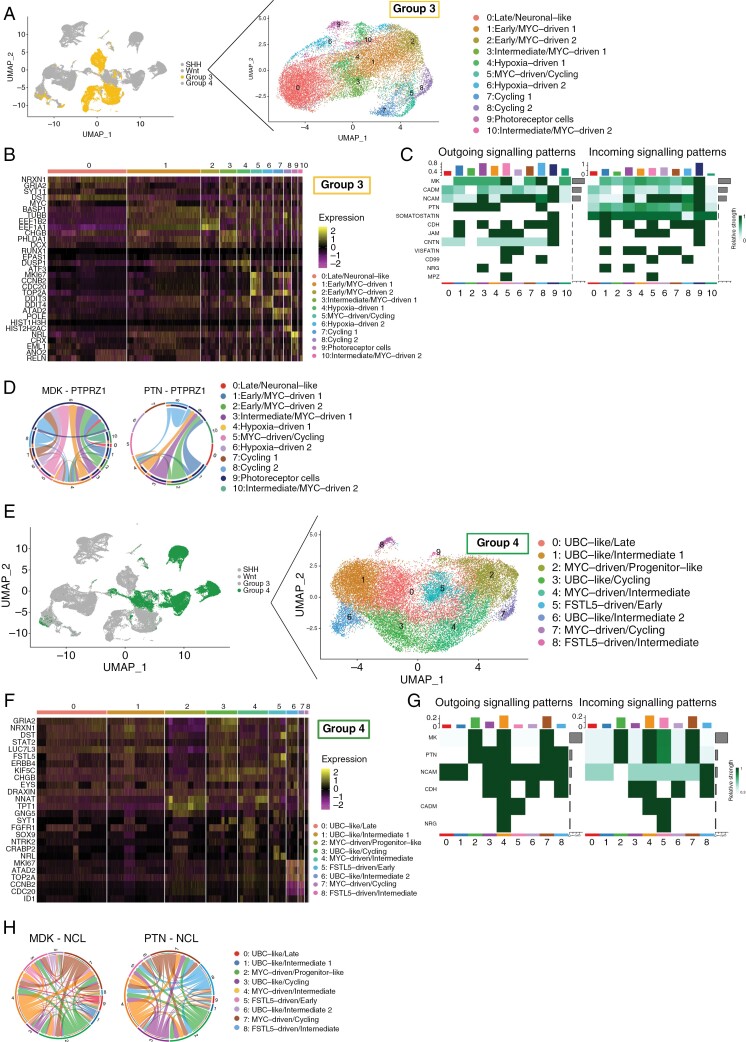
Intratumoral communications in Group 3 and Group 4 MB. Tumor cells were isolated from Group 3 (A), and Group 4 (E) medulloblastoma (MB) datasets and re-clustered yielding multiple tumor cell subpopulations. The cluster-specific markers were identified in the integrated Group 3 (*n* = 8) (B), and Group 4 (*n* = 5) (F) tumor datasets. Intratumoral signaling in Group 3 (C), and Group 4 MB (G) was analyzed using the computational tool *CellChat* and outgoing and incoming signaling was quantified based on the expression levels of ligands and their receptors, respectively. Selected significantly upregulated ligand−receptor pairs from the *CellChat* output in Group 3 (D) and Group 4 (H) MB showing cell clusters sending the signals via ligand expression and those receiving that signal via receptor expression.

We further investigated the intratumoral heterogeneity of SHH, Group 3, and Group 4 datasets (17 patients) by isolating tumor cells from immune cells and stroma and analyzing them separately (Supplementary Figure 4A, Figure 3A and E). Within the yielded 8 subclusters in the SHH tumor dataset, clusters 2, 5, and 7 contained cells that expressed early SHH MB markers, such as *SFRP1* and *HHIP*^5,26^ (Supplementary Figure 4B, Supplementary Table 3). They also expressed *HES6*—a transcriptional target of *ATOH1* expressed in the progenitors of the rhombic lip (RL) that give rise to the cerebellar granule neuron precursors (cGNPs)^27^—the cells of origin of the SHH MBs.^28^ Based on their gene expression patterns, these 3 clusters were termed “Early/Precursor-like,” “Proliferating” (due to high levels of the *MKI67* expression), and “Early differentiating,” respectively (Supplementary Figure 4B). By contrast, clusters 1, 3, 4, and 6 expressed more mature neuronal markers such as *GRIA2* (glutamate receptor)*, PAX6* (cerebellar granule cell marker), and *NRXN1* (cell adhesion molecule found on pre-synaptic membranes) and were therefore termed “Intermediate” or “Late/Neuronal-like.” Interestingly, the expression of both *SFRP1* and *HHIP*, reported to have tumor suppressor functions^29–31^—the former being the target of SHH signaling and the latter being a negative regulator of the SHH pathway—was lost in those clusters (Supplementary Figure 4B, Supplementary Table 3).

In the Group 3 integrated dataset (*n* = 8) (Figure 3A), cells expressed markers associated with ocular transcriptional programs, including *NRL*, *CRX*, and *EML1* (Figure 3B). Even though Group 3 samples were integrated from 2 different datasets, the cells clustered based on transcriptomic similarities, and not based on patient/dataset (Supplementary Figure 3A). Based on their transcriptional programs (Figure 3B, Supplementary Table 4) the Group 3 clusters were split into 3 main categories: “Early/MYC-driven” characterized by the expression of *MYC* and neural precursor genes, such as *DCX*; the “Intermediate/MYC-driven” defined by expression of *PHLDA1* and *RELN*, and the “Late/Neuronal-like” expressing genes like *NRXN1* and *GRIA2* (Figure 3B). Additionally, the Group 3 tumors contained cell populations expressing cycling genes (eg, *CCNB2*, *CDC20*), and hypoxia-related genes (eg, *DDIT3*, *DDIT4*) (Figure 3B).

The integrated Group 4 tumor datasets (*n* = 5) produced 9 clusters and represented 3 major groups: “MYC-driven,” “FSTL5-driven,” and “UBC-like” (Figure 3E) according to their transcriptional programs (Supplementary Table 5). While Group 3 tumors mainly expressed photoreceptor markers, Group 4 tumor cells expressed mainly UBC markers, including *EOMES*, *OTX2*, *NNAT*, and *LMX1A* (Figure 3F, Supplementary Figure 3B) consistent with their proposed cells of origin.^23^

### Ligand−Receptor Analysis Reveals Common Patterns in the Intratumoral Communication Within MB Subgroups

After identifying the tumor subclusters and their markers, our next step was to understand how they influence the intratumoral interactions within each MB subgroup (Figure 3, Supplementary Figure 4). Using ligand−receptor analysis tool *CellChat*^14^, we found that all 3 subgroups displayed intratumoral communication through the neural cell adhesion molecule 1 (*NCAM-1*) axis (Figure 3C and G, Supplementary Figure 4C), the cell adhesion molecule 1 (*CADM1*) axis as well as the midkine (*MDK*) pathway through the nucleolin (NCL) and PTPRZ1 ligands (Figure 3C,D,G,H, Supplementary Figure 4C and D). *NCAM-1* signaling plays an important role in neuronal cell migration, growth, and axon guidance, which has been shown to be expressed by tumor cells in gliomas and medulloblastomas, which we also observed in our SHH, Group 3, and Group 4 datasets (Supplementary Figure 5A-C). Its expression is inversely correlated with prognosis, and higher levels of *NCAM-1* have been shown to slow down the growth of these tumors.^32^

**Figure 4.**
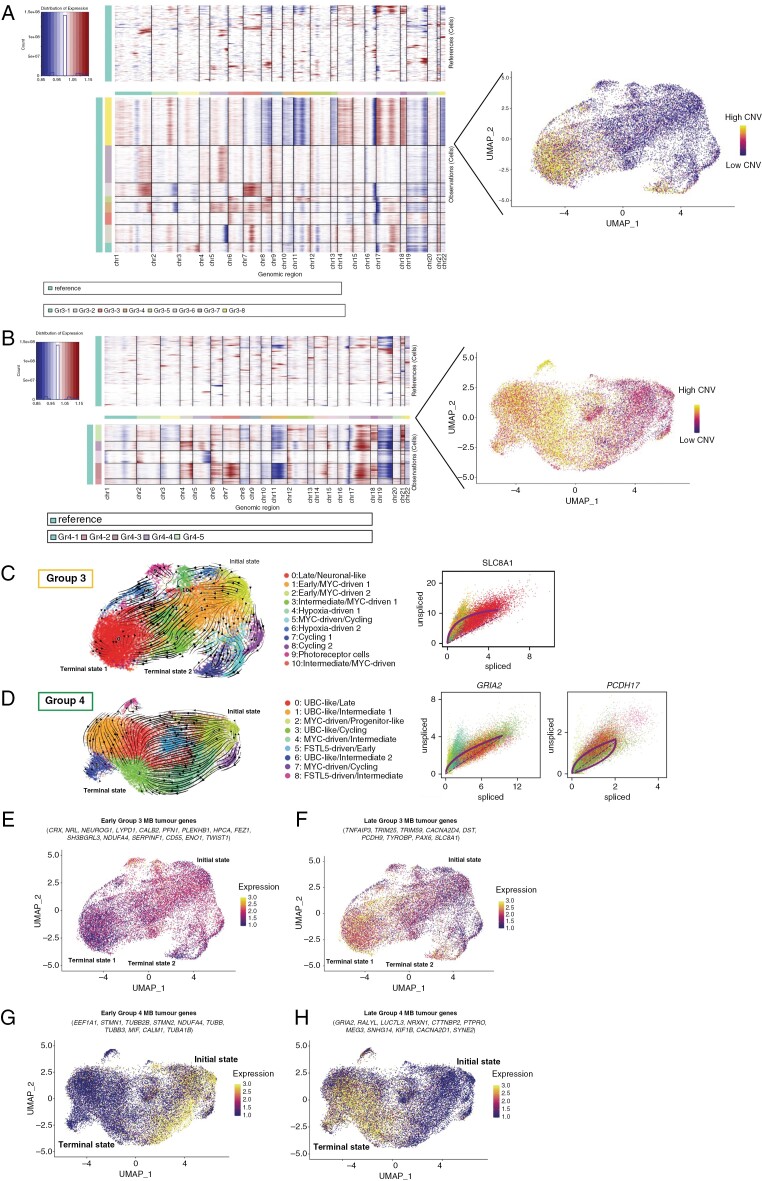
Inference of copy-number variation (CNV) levels combined with cell trajectories reveals early and late markers in medulloblastoma (MB). (A) Single-cell CNV detection using InferCNV projected onto the UMAP of integrated Group 3, and Group 4 MB samples. Average CNV levels per cell were quantified as standard deviation from the normal expression based on the reference cells (immune and stromal cells from the same patients). Cell trajectories were projected onto the UMAPs of Group 3 (B) and Group 4 (C) MB cells showing the path from the initial and terminal state(s) (left) and the driver genes (right). Early and late subgroup-specific tumor markers of Group 3 (D) and Group 4 (E) MB were then derived using CNV levels and cellular trajectories.

Notably, Group 3 MBs had the highest number of signaling pathways upregulated between different tumor cell populations, indicating a higher level of intratumoral connectivity (Figure 3C). One of the signaling pathways present only in Group 3 and Group 4 MBs was pleiotrophin (PTN) signaling (Figure 3C and G, Supplementary Figure 5D and E). Both MDK and PTN are closely related growth factors known for their pleiotropic effects on cell growth, differentiation, and survival through the activation of downstream signaling pathways including PI3K-AKT and MAPK/ERK.^33,34^ Interestingly, in Group 3 MB, the signaling was coming from several tumor clusters toward a cluster of cells expressing photoreceptor markers (Figure 3C) that also lacked CNVs (Figure 4A), therefore, resembling normal cells. Additionally, in both Group 3 and Group 4 subgroups, MDK and PTN were interacting with the same receptors −PTPRZ1 and NCL, respectively (Figure 3D and H). This analysis suggests an important role of MDK and PTN signaling in tumor proliferation in Group 3 and Group 4 MBs, presenting a potential drug target.

### CNV Levels Combined With Cell Trajectory Analysis Identify Early and Late Markers in MB Subgroups

It has been previously demonstrated that CNV levels affect clustering in MB.^5^ This was validated for every MB subgroup in our dataset (Figure 4A and B, Supplementary Figure 6A). However, we wanted to understand if the CNV levels were reflective of different cell states in the course of tumor progression as this would potentially allow us to derive cell-state-specific markers. For this, we performed RNA velocity analysis^6^ and projected the cell trajectories of individual cells onto the UMAPs of each MB subgroup within our dataset (Figure 4 C and D, Supplementary Figure 6B). We found that indeed the directionality of cell trajectories was moving from those with low to those with high CNV levels pointing to earlier and later cell states within those tumors. These trajectories were also consistent with our cluster annotation, where “earlier” clusters expressed neuronal precursor/progenitor markers, whereas “later” clusters expressed mature neuronal markers, as described earlier.

We used the dynamical model of the RNA velocity analysis^17^ to identify the driver genes and the initial and terminal states in each subgroup (Figure 4 C and D, Supplementary Figure 6B). Interestingly, SHH tumors had 2 terminal states: terminal state 1 ending at the proliferative cluster 3 expressing *MKI67* and terminal state 2 at cluster 1 expressing mature neuronal markers such as *GRIA2* and *NRXN1* (Supplementary Figure 7A). *NRXN1* was the main gene driving the trajectory towards terminal state 2 (Supplementary Figure 6B) suggesting different driving programs within these tumors. Group 3 tumors also had 2 terminal states, which were influenced by the cell cycle genes (Figure 4C). Cluster 7, identified as terminal state 2, expressed *TOP2A*, *MKI67*, and *E2F1*, whereas cluster 0 expressed mature neuronal markers similar to those of cluster 1 in SHH tumors including *GRIA2* and *NRXN1* (Supplementary Figure 7B). In contrast with SHH and Group 3 tumors, Group 4 tumors showed a pronounced unidirectionality of its cellular trajectories (Figure 4D) from cluster 2 expressing neural-specific developmental genes such as *NNAT*, *SIRT2*, and *STMN1* to cluster 3, expressing mature neuronal markers including *GRIA2* (Supplementary Figure 7C).

After discovering that the cellular trajectories move from cells with low CNV levels to those with high CNV levels, we termed the initial state clusters as “early” tumor cells, and those at the terminal states as “late” tumor cells. We then performed a DGE analysis between them to identify early and late markers, respectively. In SHH tumors, several early markers were related to transcriptional targets downstream of the SHH signaling pathway, including *SFRP1* and *SFRP5*,^35,36^ as well as *SOX2* and *CD24* − both markers of neural progenitor cells^37^ with *SOX2* being responsible for the maintenance of stemness and pluripotency of embryonic stem cells in normal development^38^ (Supplementary Figure 6C). Additionally, early SHH tumor cells showed a high expression of *ATOH1*—a transcription factor responsible for the fate specification of the progenitors of the RL.^27^ Late SHH markers mainly included those expressed in mature neurons such as *GRIA2* and *NRXN1* (Supplementary Figure 6C). Most genes identified as early Group 3 markers were those involved in retinal neuron growth and differentiation including *CRX*, *NRL*, and *NEUROG1* (Figure 4E). Both NRL and CRX have been shown to be required for tumor maintenance in Group 3 MB^39^ as well as defining Group 3 tumor identity.^40^ Additionally, *ENO1* which has been shown to promote tumorigenesis in multiple types of cancer through the activation of the PI3K/AKT pathway,^41^ was upregulated in early Group 3 tumor cells. One of the late Group 3 markers—*SLC8A1*—was also identified as the main driver gene of cellular trajectories in this MB subgroup (Figure 4F). Early markers for Group 4 tumors included tubulins (*TUBB*, *TUBB3*, *TUBB2B*, *TUBA1A*) and stathmins *STMN1* and *STMN2* (Figure 4G). Stathmins play an important role in the regulation of the cytoskeleton with *STMN2* expression being specific to neurons and required for the growth and maintenance of the neuronal axons.^42^*STMN1*, on the other hand, is expressed ubiquitously but is also known for its oncogenic properties by increasing the rate of cellular proliferation, and is associated with worse prognosis in MB.^43^ Stathmins regulate the cytoskeleton by interacting with the microtubules inside the cells and promoting their depolymerization, thus allowing the cells to divide.^44^ High expression of several tubulin and stathmin genes in our Group 4 dataset suggests activation of cell proliferation at earlier stages (ie, prior to the accumulation of the CNVs) and could potentially be targeted with novel anti-tubulin therapies. The later Group 4 markers, as we mentioned earlier, included more mature neuronal markers such as *GRIA2* and *NRXN1* (Figure 4H).

### Heterogenous Patterns of Accumulated CNVs Underlie Tumor Evolution in Group 3 and Group 4 MB

**Figure 5.**
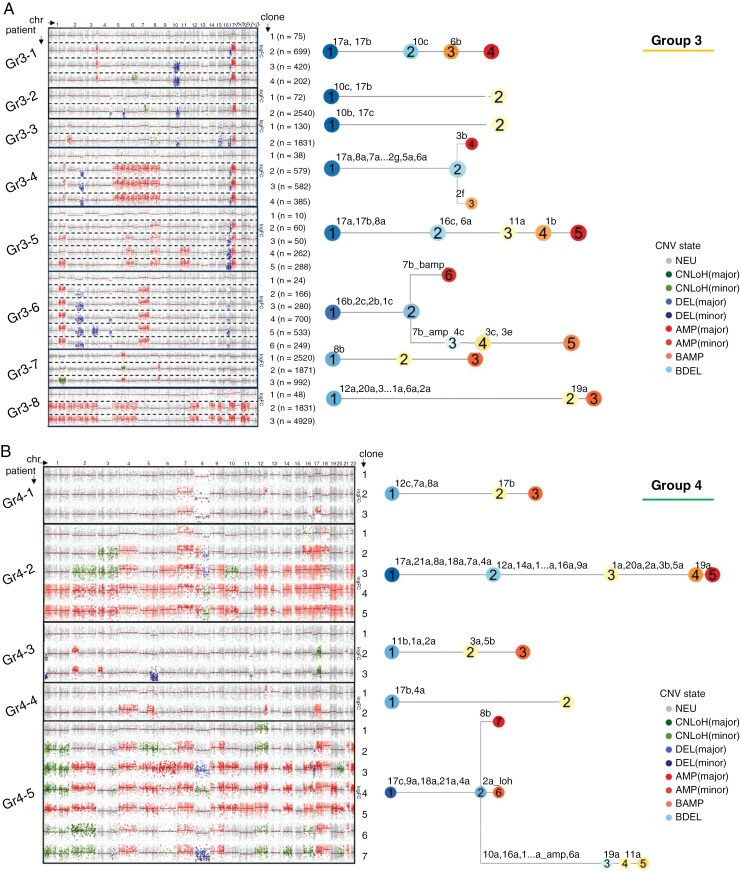
Clonal evolution of Group 3 and Group 4 MB tumors. (A, B) Copy-number variation (CNV) in individual clones of the 8 Group 3 MB tumors (Gr3-1–Gr3-8) (A) and 5 Group 4 tumors (Gr4-1–Gr4-5) (B) shown as a heatmap with chromosomes (chr) as columns and clones as rows. Number of cells per clone are specified in brackets. Black lines separate the patients. Every CNV gained in each new clone is shown in each tumor’s phylogenetic tree with a chromosome number and the section of the chromosome affected (a, b, c, etc.).

Prompted by our findings in early and late cell populations, we next investigated the clonal architecture of medulloblastoma tumors. For this analysis, we used a newly developed computational tool called *Numbat*^7^ that infers CNVs in single cells by combining the expression, allele, and haplotype information derived from the population-based allele phasing. Utilizing allele and haplotype information in addition to the expression levels allows for a more accurate prediction of consecutive CNV gains, which in turn allowed us to track the clonal evolution within each individual tumor. After analyzing the SHH, Group 3, and Group 4 tumor datasets individually, we found that, interestingly, SHH tumors consisted only of 2 clones (Supplementary Figure 8). Furthermore, the SHH-derived tumor clones had very similar CNV patterns, including previously described gains of chr 3 and 9p, as well as losses on chr 9q, 10q, and 17^45^ (Supplementary Figure 8). For this analysis, we therefore focused mainly on the Group 3 and Group 4 tumors that had much more variable genomic landscapes (Figure 5). We managed to identify individual clones in every tumor dataset characterized by specific CNVs and reconstruct their phylogenetic trees (Figure 5A and B). As opposed to the expression-based methods, including *InferCNV*, that can only identify chromosomal gains and losses, *Numbat* is superior in identifying copy-neutral loss-of-heterozygosity (CNLoH), that would lead to a downregulation of gene expression, and the downregulation of protein expression for which 2 copies of the gene are required, without detecting that one of the chromosomal regions has been lost. Once we had identified the clones, we then performed a DGE analysis to detect the clone-specific markers. It is of note that cells from the same clones do not necessarily cluster together on a UMAP, since the progression from one clone to the next can be characterized by only one genomic change and is therefore not enough to create the transcriptomic differences necessary for the clustering. However, since the analysis of the individual clones unveils a greater level of detail, we wanted to understand whether similar patterns could emerge between the Group 3 and the Group 4 tumors and whether any previously undescribed markers of tumor progression could be identified in medulloblastoma.

### SOX4 Expression Increases in Later Tumor Clones in Group 3 and Group 4 MB

By analyzing the CNV patterns in every dataset, we found that every Group 3 and Group 4 tumor was characterized by a unique genomic landscape, and very few genomic aberrations were shared between tumors (Figure 5A and B). The most common CNVs within Group 3 tumors were a gain of 17q, in some cases combined with a 17p loss, as well as a 1q gain (Figure 5A). In Group 4 a gain of chr 7, 17, and 18, as well as a chr 8 loss were amongst the most common (Figure 5B).

**Figure 6.**
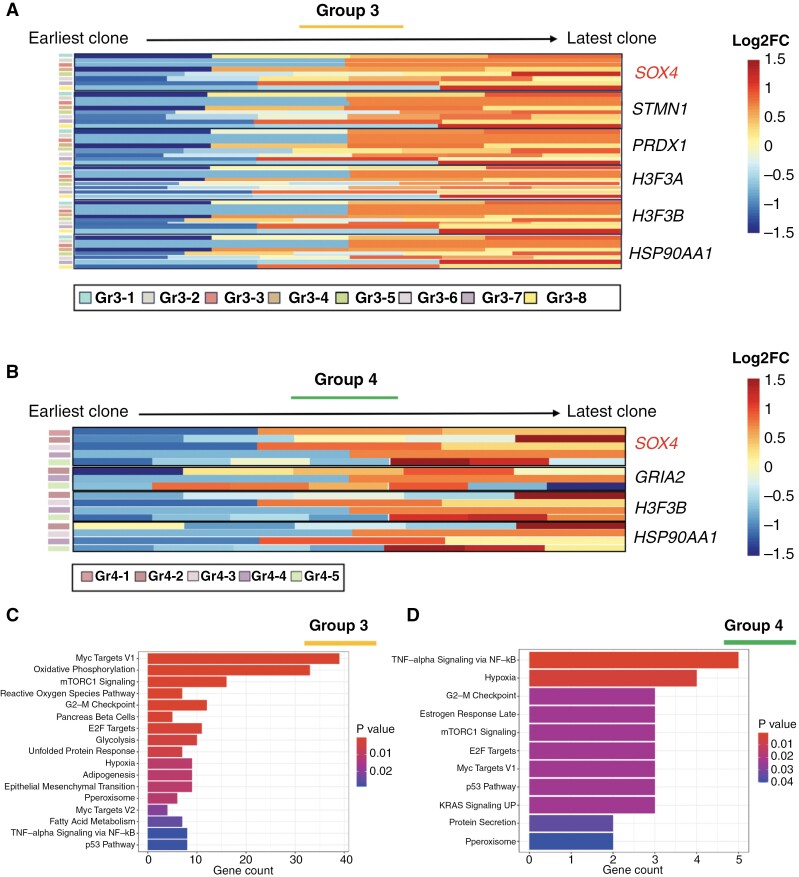
Group 3 and Group 4 medulloblastoma (MB) tumors show transcriptomic similarities in the progression from earlier to later clones. DEG between earlier and later clones identified through *Numbat* in Group 3 (A) and Group 4 (B) tumors. Significantly enriched signaling pathways based on DEG between individual clones in Group 3 (C) and Group 4 (D) tumors. AMP = amplification; BAMP = balanced amplification; BDEL = balanced deletion; chr = chromosome; CNLoH = copy-neutral loss-of-heterozygosity; CNV = copy-number variation; DEL = deletion; NEU = neutral.

Despite the absence of common patterns that would extend to every tumor in these 2 subgroups, we identified important similarities in the gene expression changes from the earlier to the later clones. The evolution of individual tumor clones from “earlier” to “later” was defined by the consecutive accumulation of CNVs where each newly gained CNV marked a new clone (Figure 5A and B). By using DESeq2^21^ for our DGE analysis, we discovered that every tumor in both Group 3 and Group 4 datasets showed an upregulation of the *SOX4* gene in the later clones, with 10 out of 13 tumors showing a statistically significant upregulation (*P*_adj_ < .05) (Figure 6A and B). *SOX4* is a transcription factor, which, during normal development, plays many important roles in stemness, differentiation, and cell fate.^46^ However, it has also been described as an oncogene that has been shown to be upregulated in more than 20 different types of cancers.^8^ In our dataset, combined with that from Riemondy et al.,^5^*SOX4* was strikingly the only gene that was identified as a significant differentially expressed marker between earlier and later clones that was common in all Group 3 and Group 4 patients (Figure 6A and B, Supplementary Figure 9).

Finally, we identified the *HSP90AA1* gene being upregulated in later clones in 12 out of 13 Group 3 and Group 4 patients (Figure 6A and B). *HSP90AA1* encodes a heat shock protein 90kDa alpha, member A1 protein—an isoform of a stress-induced HSP90 protein. It has been shown that HSP90 is a potential target in MYC-driven medulloblastomas, where treatment with an HSP90 inhibitor Onalespib significantly reduced the growth of those tumors and prolonged the survival in mice.^47^ Its upregulation in later clones in our tumor datasets further indicates its importance for the progression of human MYC-driven tumors and suggests its relevance as a therapeutic target. In addition to identifying clone-specific markers, we also identified several differentially activated signaling pathways in both MB subgroups (Figure 6C and D). As expected, we found an enrichment of Myc targets V1 pathway in both Group 3 and Group 4 tumor clones, as well as mTORC1 and KRAS signaling pathways indicating increased proliferative properties in later tumor clones.

## Discussion

In this study, we described the pathways of intratumoral communication, identified early and late markers in MB subgroups, and described the evolutionary trajectories and clone-specific transcriptional programs for Group 3 and Group 4 MBs. In SHH, Group 3 and Group 4 MBs, we identified upregulated *NCAM-1* and *MDK* signaling as facilitators for communication between different tumor clusters. Despite *NCAM-1* expression having an inverse correlation with prognosis, it has been shown to have a positive correlation with tumor mutational burden, which is an observation consistent with our findings.^32^ It was suggested that higher mutation levels attracted more immune cells—particularly NK cells expressing *NCAM-1*, thus leading to a better prognosis. However, since we observed activation of tumor-derived *NCAM-1* signaling in Group 3 tumors, which are known for being “immunologically cold,”^48^ we suggest that *NCAM-1* signaling occurs within different cellular compartments depending on MB subgroup. Of note, NCAM-1 upregulation has been correlated with poor prognosis in other types of cancers, including gliomas.^49^ In SHH and Group 4 MBs, the interaction within the MK signaling pathway occurred between the MDK ligand and the NCL receptor, and in both MB subgroups the outgoing signaling derived from cells with less mature neuronal markers. MDK signaling has been shown to promote invasion and metastasis in multiple types of cancer^50^ thus presenting a potential therapeutic target.

One of the main findings of our study was the fact that MB tumors display unique clonal and genomic landscapes and very few common CNVs. These differences further underline the need for personalized treatments and provide potential explanations for treatment resistance and relapse, since the choice of clinical treatment protocol is based solely upon subgroup and risk stratification. Additionally, according to the 2021 WHO classification of tumors of the CNS, MB tumors have been subdivided further, including 4 subtypes of SHH MB, and 8 subtypes of Group 3/Group 4 MB.^51^ However, it has been pointed out that stratification of these tumors into groups that are too small carries a risk that appropriate studies cannot be designed for them, and that in order to identify relevant therapeutic targets it is important to focus on molecular similarities that these tumors share.^52^ Therefore, in our study, we focused on finding the common targets, especially for Group 3/Group 4 tumors that comprise the majority of MBs. Despite their molecular, genomic, and prognostic differences, we managed to identify *SOX4* as a marker of the late tumor clones shared between both Group 3 and Group 4 subgroups. Targeting *SOX4* therapeutically could potentially prevent the tumor progression towards later clones, but further research is needed since SOX4, being a transcription factor, poses further challenges and risks of off-target effects. We also detected the upregulation of heat shock proteins in the Group 3 tumors, the inhibition of which has been shown to be effective in slowing down tumor progression in mice and can therefore potentially be translated into the clinical practice.^47^

Despite having relatively good survival rates, the outcome of medulloblastoma largely depends on its subgroup, the disease burden at diagnosis as well as disease recurrence. In addition, current treatment methods come with severe side effects that need to be addressed. Therefore, we believe that a further deconvolution of the medulloblastoma tumor cell heterogeneity and proposed therapeutic targets presented in this study is a step forward towards more personalized and less harmful forms of treatments for these patients.

## Supplementary Material

vdae172_suppl_Supplementary_Figures

vdae172_suppl_Supplementary_Table_S1

vdae172_suppl_Supplementary_Table_S2

vdae172_suppl_Supplementary_Table_S3

vdae172_suppl_Supplementary_Table_S4

vdae172_suppl_Supplementary_Table_S5

## Data Availability

The single-cell data will be made available upon request and the code will be published in a GitHub repository.
