# Supplementary material for: Genomic tumor evolution dictates human medulloblastoma progression
Source: Neurooncol Adv. 2024 Oct 5;6(1):vdae172. doi: 10.1093/noajnl/vdae172 (PMC11629688; doi:10.1093/noajnl/vdae172)
Supplement: vdae172_suppl_Supplementary_Figures [file vdae172_suppl_supplementary_figures.pdf]

A

Copy number variation profile

SHH-1

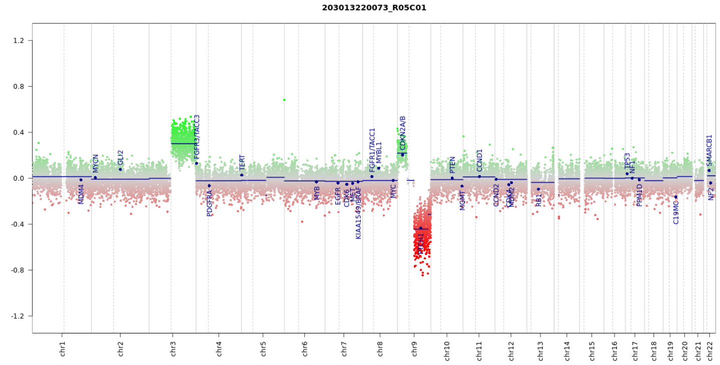

Depiction of chromosome 1 to 22 (and X/Y if automatic prediction was successful). Gains/ amplifications represent positive, losses negative deviations from the baseline. 29 brain tumor relevant gene regions are highlighted for easier assessment. (see Hovestadt & Zapatka, http://www.bioconductor.org/packages/devel/bioc/html/conumee.html)

MGMT promotor methylation (MGMT-STP27)

MGMT promotor status prediction

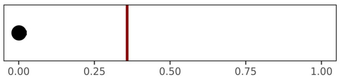

Status	Estimated	CI lower	CI upper
unmethylated	0.00089	0.00004	0.02212

(see Bady et al, J Mol Diagn 2016; 18(3):350-61)

Copy number variation profile

SHH-2

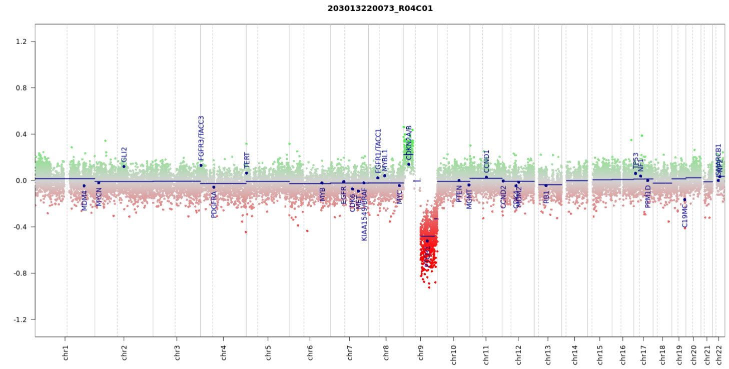

Depiction of chromosome 1 to 22 (and X/Y if automatic prediction was successful). Gains/ amplifications represent positive, losses negative deviations from the baseline. 29 brain tumor relevant gene regions are highlighted for easier assessment. (see Hovestadt & Zapatka, http://www.bioconductor.org/packages/devel/bioc/html/conumee.html)

MGMT promotor methylation (MGMT-STP27)

MGMT promotor status prediction

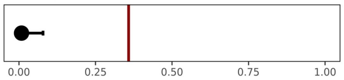

Status	Estimated	CI lower	CI upper
unmethylated	0.00826	0.00081	0.07860

(see Bady et al, J Mol Diagn 2016; 18(3):350-61)

Copy number variation profile

SHH-3

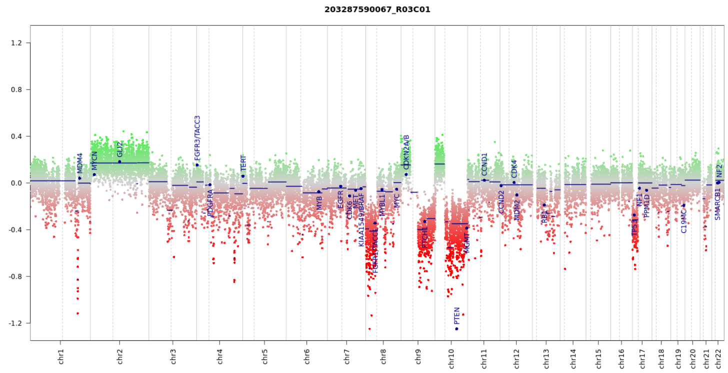

Depiction of chromosome 1 to 22 (and X/Y if automatic prediction was successful). Gains/ amplifications represent positive, losses negative deviations from the baseline. 29 brain tumor relevant gene regions are highlighted for easier assessment. (see Hovestadt & Zapatka, http://www.bioconductor.org/packages/devel/bioc/html/conumee.html)

MGMT promotor methylation (MGMT-STP27)

MGMT promotor status prediction

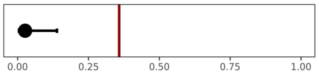

Status	Estimated	CI lower	CI upper
unmethylated	0.02627	0.00450	0.13876

(see Bady et al, J Mol Diagn 2016; 18(3):350-61)

Copy number variation profile

SHH-4

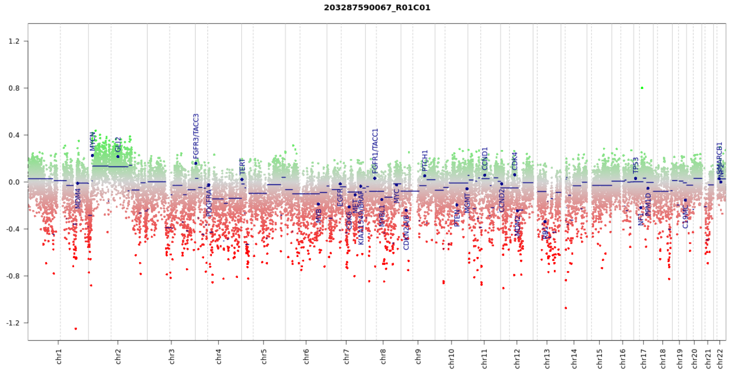

Depiction of chromosome 1 to 22 (and X/Y if automatic prediction was successful). Gains/ amplifications represent positive, losses negative deviations from the baseline. 29 brain tumor relevant gene regions are highlighted for easier assessment. (see Hovestadt & Zapatka, http://www.bioconductor.org/packages/devel/bioc/html/conumee.html)

MGMT promotor methylation (MGMT-STP27)

MGMT promotor status prediction

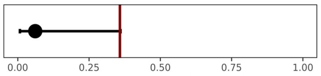

Status	Estimated	CI lower	CI upper
u nmethylated	0.06116	0.00748	0.36029

(see Bady et al, J Mol Diagn 2016; 18(3):350-61)

B

Analysis results

Wnt-1

**Workflow name:**	brain_classifier_v11b4_sample_report
**Workflow version:**	3.2
**Workflow identifier:**	Workflow_124_12=>201465850002_R03C01|15=>NM2_0454-1-DNA-T.v1_1R03C01|16=>DNA-KRYO|17=>NA|18=>CNS
**Run started:**	2022-02-24T15:51:25.000000Z
**Workflow description**	Complete analysis with brain classifier version v11b4 (including v12.5) with sample identifier

Classification using methylation profiling is a research tool under development, it is not verified and has not been clinically validated. Implementation of the results in a clinical setting is in the sole responsibility of the treating physician. This tool/website is not HIPAA compliant.

Tasks

Copy number prediction

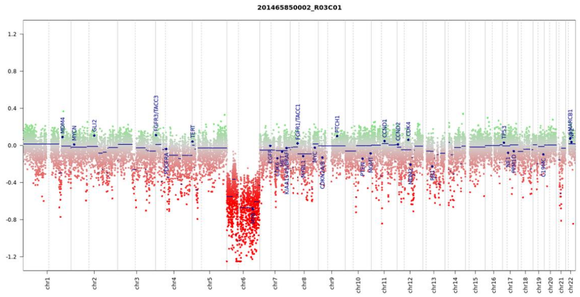

Depiction of chromosome 1 to 22 (and X/Y if automatic prediction was successful). Gains/amplifications represent positive, losses negative deviations from the baseline. 29 brain tumor relevant gene regions are highlighted for easier assessment.

(see Hovestadt & Zapatka, http://www.bioconductor.org/packages/devel/bioc/html/conumee.html

Classifier prediction (Brain tumor classifier; Version: 12.5)

Copy number variation profile

Wnt-2

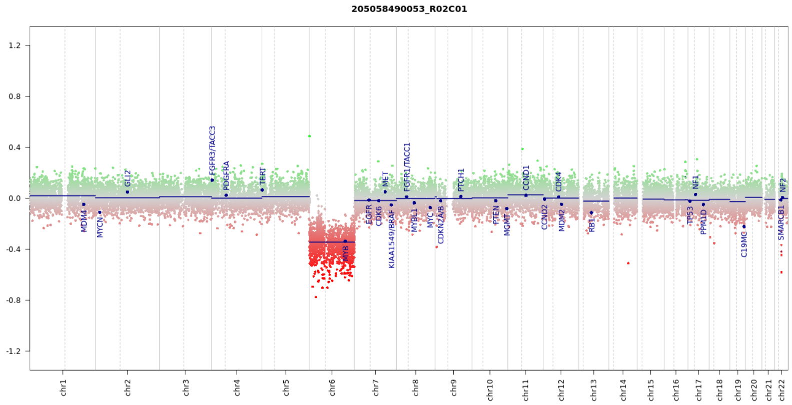

Depiction of chromosome 1 to 22 (and X/Y if automatic prediction was successful). Gains/amplifications represent positive, losses negative deviations from the baseline. 29 brain tumor relevant gene regions are highlighted for easier assessment. (see Hovestadt & Zapatka, http://www.bioconductor.org/packages/devel/bioc/html/conumee.html)

MGMT promotor methylation (MGMT-STP27)

MGMT promotor status prediction

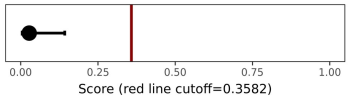

Status	Estimated	CI lower	CI upper
unmethylated	0.02748	0.00478	0.14251

(see Bady et al, J Mol Diagn 2016; 18(3):350-61)

Copy number variation profile

Wnt-3

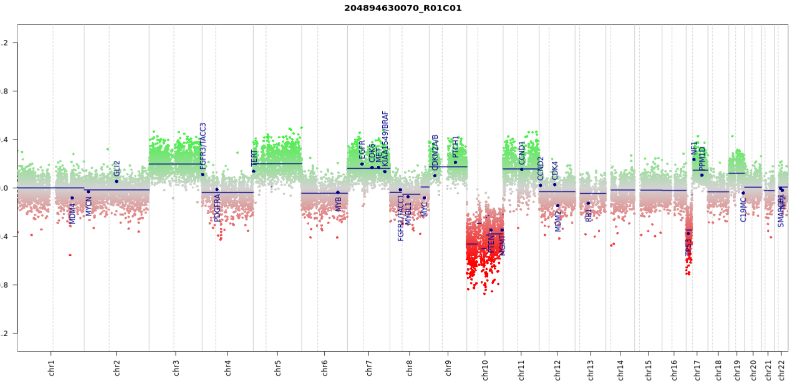

MGMT promotor status prediction

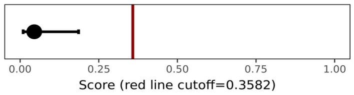

Status	Estimated	CI lower	CI upper
unmethylated	0.04484	0.00957	0.18578

(see Bady et al, J Mol Diagn 2016; 18(3):350-61)

C

Copy number variation profile — Gr3-1

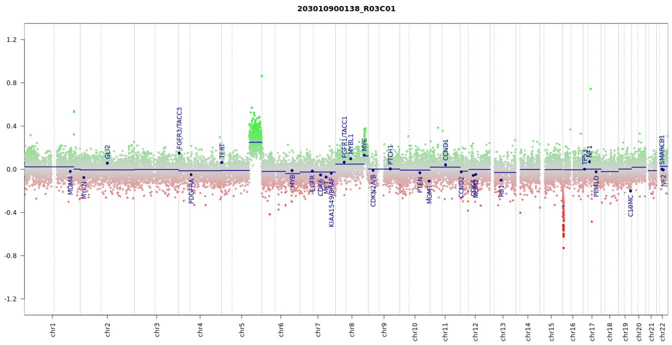

Depiction of chromosome 1 to 22 (and X/Y if automatic prediction was successful). Gains/ amplifications represent positive, losses negative deviations from the baseline. 29 brain tumor relevant gene regions are highlighted for easier assessment. (see Hovestadt & Zapatka, http://www.bioconductor.org/packages/devel/bioc/html/conumee.html)

MGMT promotor methylation (MGMT-STP27)

MGMT promotor status prediction

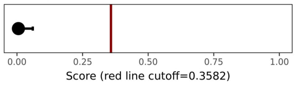

Status	Estimated	CI lower	CI upper
unmethylated 0.00541		0.00046	0.06028

(see Bady et al, J Mol Diagn 2016; 18(3):350-61)

Copy number variation profile — Gr3-2

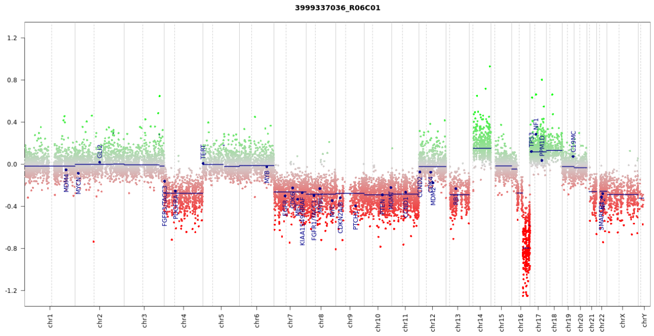

Depiction of chromosome 1 to 22 (and X/Y if automatic prediction was successful). Gains/ amplifications represent positive, losses negative deviations from the baseline. 29 brain tumor relevant gene regions are highlighted for easier assessment. (see Hovestadt & Zapatka, http://www.bioconductor.org/packages/devel/bioc/html/conumee.html)

MGMT promotor methylation (MGMT-STP27)

MGMT promotor status prediction

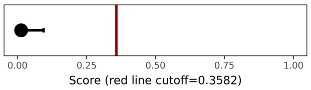

Status	Estimated	CI lower	CI upper
unmethylated 0.01303		0.00167	0.09441

(see Bady et al, J Mol Diagn 2016; 18(3):350-61)

D

Copy number variation profile Gr4-1

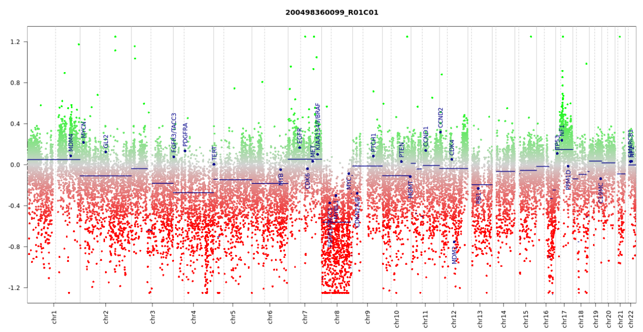

Depiction of chromosome 1 to 22 (and X/Y if automatic prediction was successful). Gains/amplifications represent positive, losses negative deviations from the baseline. 29 brain tumor relevant gene regions are highlighted for easier assessment. (see Hovestadt & Zapatka, http://www.bioconductor.org/packages/devel/bioc/html/conumee.html)

MGMT promotor methylation (MGMT-STP27)

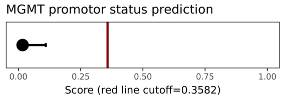

Statu s	Estimate d	CI lower	CI upper
unmethylate d	0.0167 7	0.0023 5	0.1097 9

(see Bady et al, J Mol Diagn 2016; 18(3):350-61)

Copy number variation profile Gr4-2

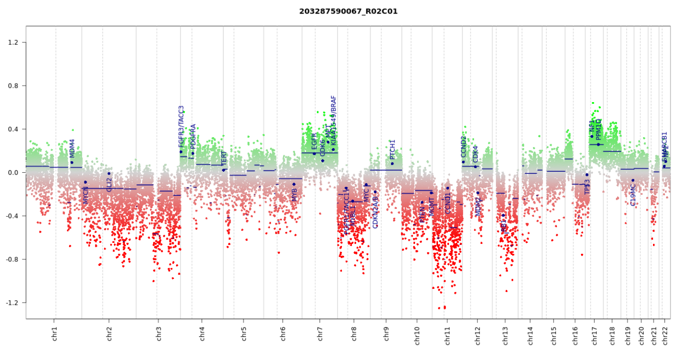

Depiction of chromosome 1 to 22 (and X/Y if automatic prediction was successful). Gains/amplifications represent positive, losses negative deviations from the baseline. 29 brain tumor relevant gene regions are highlighted for easier assessment. (see Hovestadt & Zapatka, http://www.bioconductor.org/packages/devel/bioc/html/conumee.html)

MGMT promotor methylation (MGMT-STP27)

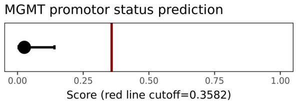

Statu s	Estimate d	CI lower	CI upper
unmethylate d	0.0260 6	0.0043 6	0.1406 2

(see Bady et al, J Mol Diagn 2016; 18(3):350-61)

Copy number variation profile Gr4-3

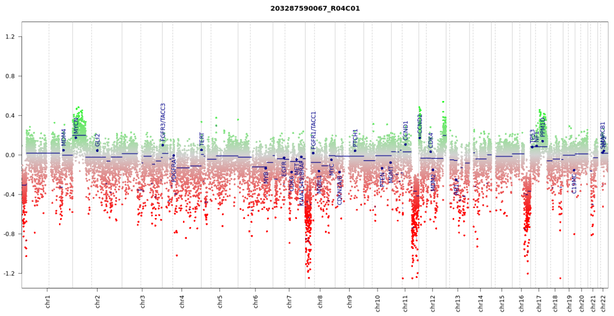

Depiction of chromosome 1 to 22 (and X/Y if automatic prediction was successful). Gains/amplifications represent positive, losses negative deviations from the baseline. 29 brain tumor relevant gene regions are highlighted for easier assessment. (see Hovestadt & Zapatka, http://www.bioconductor.org/packages/devel/bioc/html/conumee.html)

MGMT promotor methylation (MGMT-STP27)

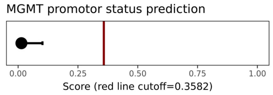

Sta tus	E st imate d	CI lowe r	CI uppe r
unmethylated	0.01 3 7 0	0 .00 17	0 .1 01 7

(see Bady et al, J Mol Diagn 2016; 18(3):350-61)

Copy number variation profile Gr4-4

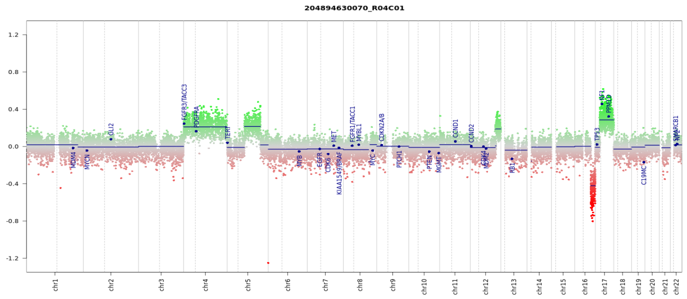

Depiction of chromosome 1 to 22 (and X/Y if automatic prediction was successful). Gains/amplifications represent positive, losses negative deviations from the baseline. 29 brain tumor relevant gene regions are highlighted for easier assessment. (see Hovestadt & Zapatka, http://www.bioconductor.org/packages/devel/bioc/html/conumee.html)

MGMT promotor methylation (MGMT-STP27)

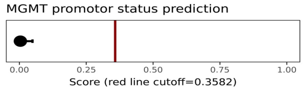

Sta tus	E st imate d	CI lowe r	CI uppe r
unmethylate d	0 .0 0 3 7 2	0 .0 0 0 2 7	0 .0 4 9 0 0

(see Bady et al, J Mol Diagn 2016; 18(3):350-61)

Copy number variation profile Gr4-5

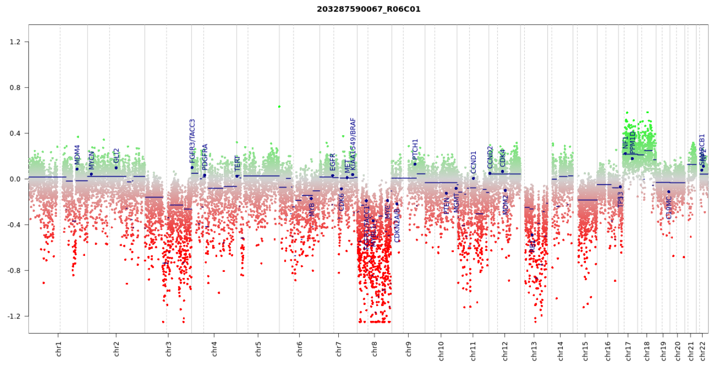

Depiction of chromosome 1 to 22 (and X/Y if automatic prediction was successful). Gains/amplifications represent positive, losses negative deviations from the baseline. 29 brain tumor relevant gene regions are highlighted for easier assessment. (see Hovestadt & Zapatka, http://www.bioconductor.org/packages/devel/bioc/html/conumee.html)

MGMT promotor methylation (MGMT-STP27)

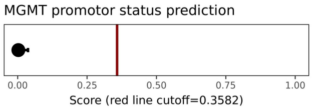

Status	E st imated	CI lower	CI upper
unmethylated	0.00243	0.0001 5	0.0372 9

(see Bady et al, J Mol Diagn 2016; 18(3):350-61)

Suppl. Fig 1. Copy number variation profiles of 14 MB tumours inferred from the methylation data. Copy number variations were inferred from DNA methylation statuses using Heidelberg pipeline of various genes in all 14 MB patients from all 4 MB subtypes: SHH (**A**), Wnt (**B**), Group 3 (**C**), and Group 4 (**D**).

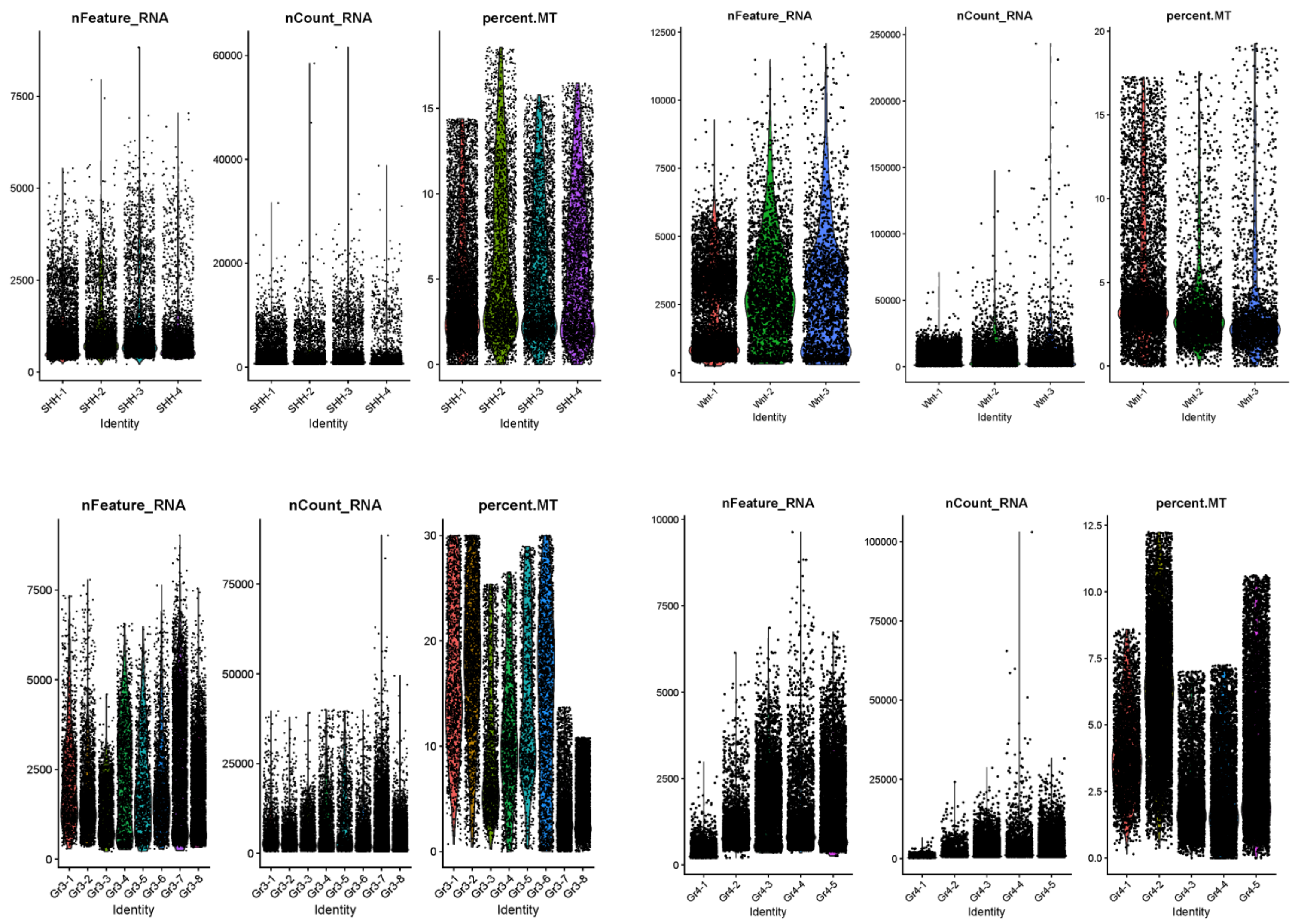

Suppl. Fig 2. Quality control of individual patient samples. Quality control was performed on each sample in which the number of gene counts (nFeature_RNA), RNA counts (nCount_RNA), and percentage of mitochondrial genes (percent.MT) were calculated. In order to filter out low-quality cells a threshold of a minimum gene count of 200 genes per cell was chosen. The cells where the mitochondrial counts exceeded the sample median percent.MT + 2x standard deviation were also excluded to filter out dead cells.

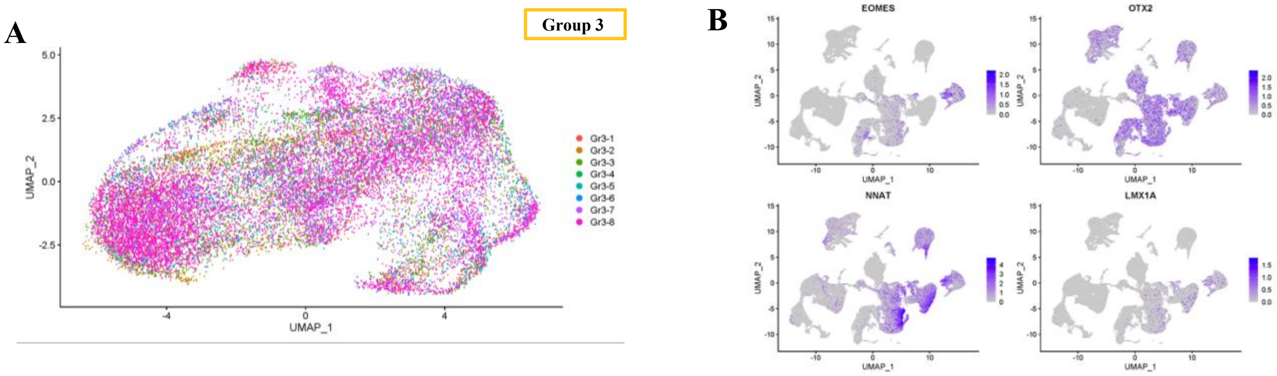

Suppl. Fig 3. Re-clustering of Group 3 and Group 4 MB tumour subtypes. A: Following the integration of our dataset with that of Riemondy et al., 2022, the clusters within the integrated object were determined and visualised using a UMAP. No batch effect was seen between the two Group 3 datasets, and instead, the cells clustered according to the similarities in their transcriptomes. **B**: Single cells from all MB subtypes projected onto one UMAP (see **Fig 1B**) with unipolar brush cell (UBC) signature upregulated in Group 4 MB cells.

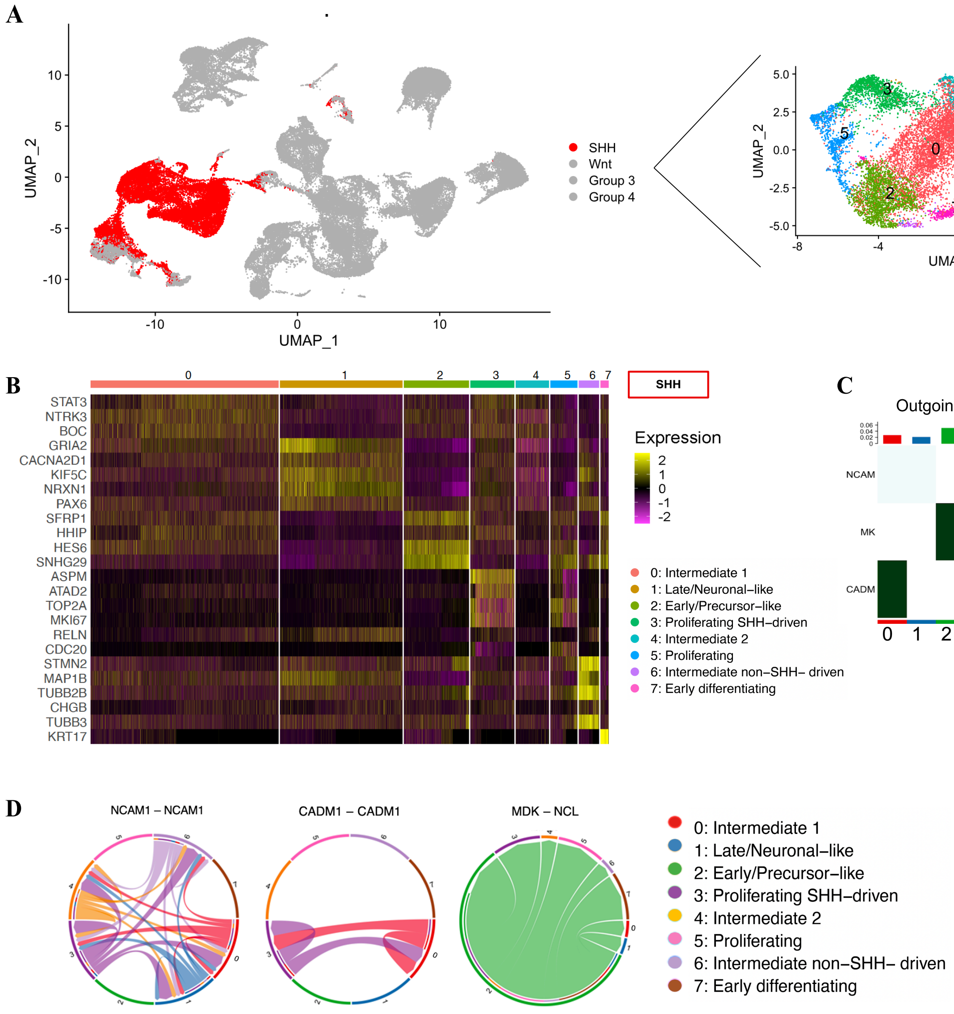

Suppl. Fig. 4. Intratumoral communication in SHH MB. A: Tumour cells were isolated from an integrated SHH MB dataset (n=4) and re-clustered yielding multiple tumour cell subpopulations. **B**: Cluster-specific markers of integrated SHH tumour dataset. **C**: Intra-tumoral signalling was analysed using the computational tool *CellChat* and outgoing and incoming signalling was quantified based on the expression levels of ligands and their receptors, respectively. **D**: Selected significantly upregulated ligand-receptor pairs from the *CellChat* output showing cell clusters sending the signals via ligand expression and those receiving that signal via receptor expression.

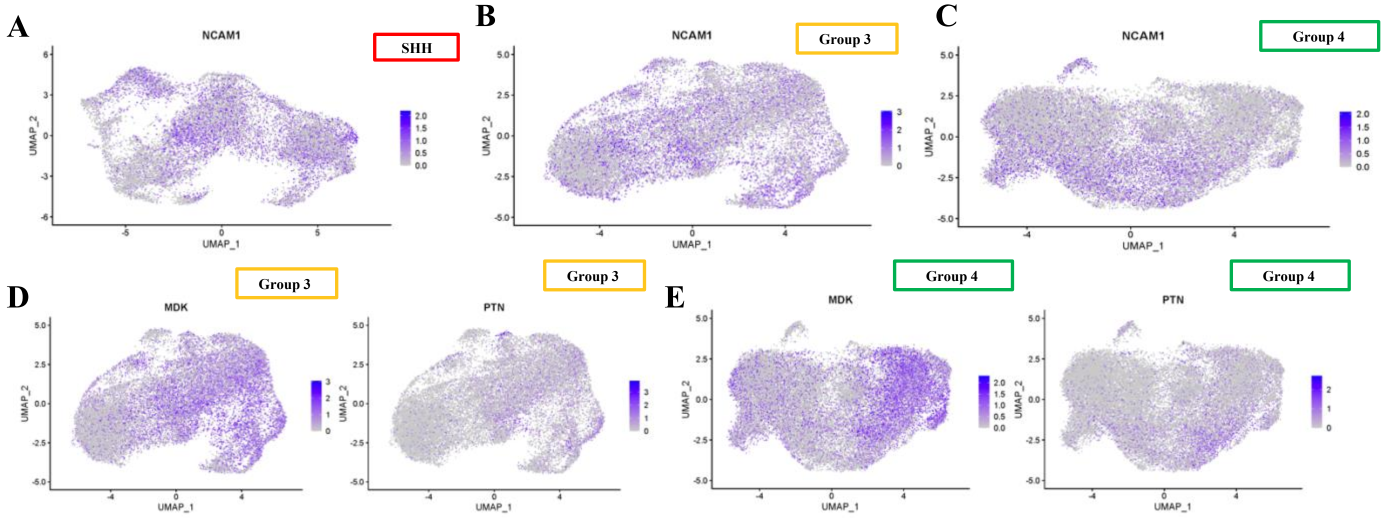

Suppl. Fig 5. Expression patterns of selected ligands in medulloblastoma. The expression levels of *NCAM1*, *MDKI*, and *PTN* from significantly upregulated signalling pathways identified using *CellChat* were plotted onto the UMAPs of the integrated SHH (**A**), Group 3 (**B, D**), and Group 4 datasets (**C, E**).

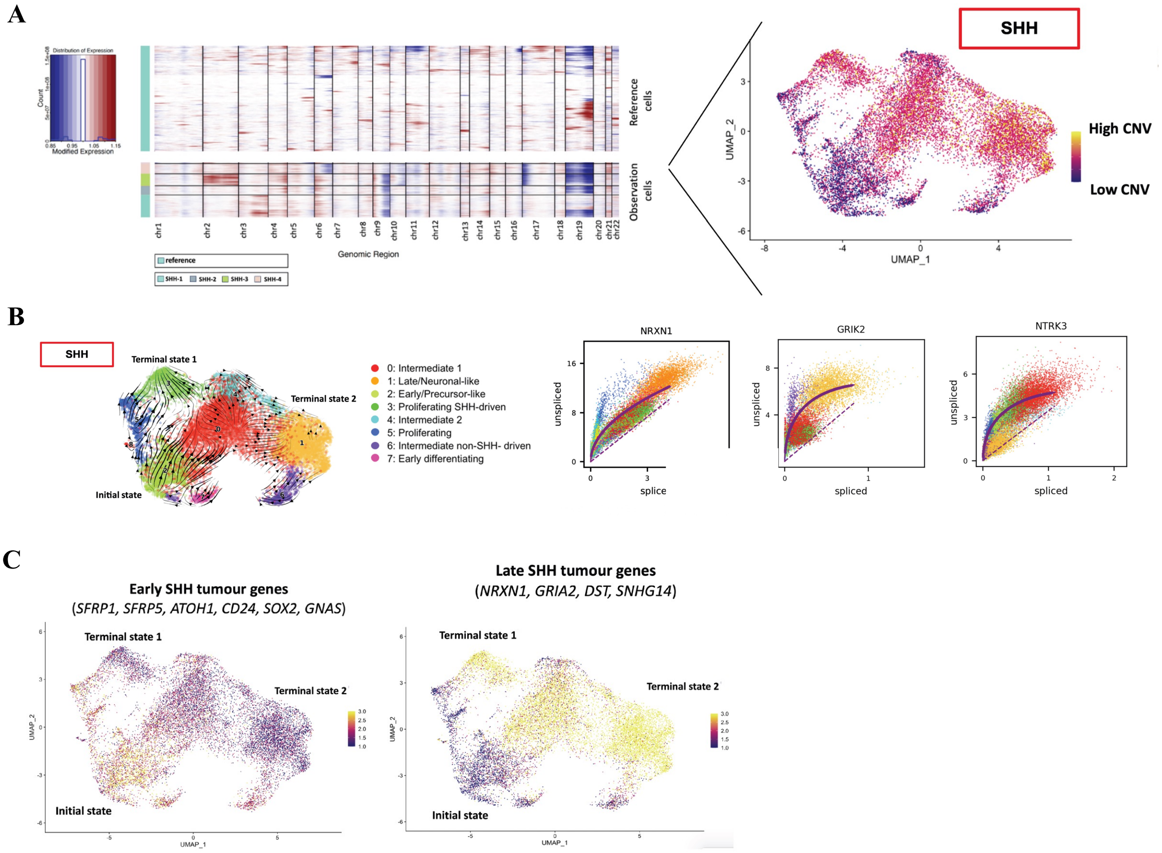

Suppl. Fig. 6. Inference of CNV levels combined with cell trajectories reveals early and late markers in SHH MB. **A**: Single-cell CNV detection using InferCNV projected onto the UMAP of integrated SHH MB samples. Average CNV levels per cell were quantified as standard deviation from the normal expression based on the reference cells (immune and stromal cells from the same patients). **B**: Cell trajectories were projected onto the UMAPs of SHH MB cells showing the path from the initial and terminal states (left) and the driver genes (right). **C**: Early and late subgroup-specific tumour markers of SHH MB were then derived using CNV levels and cellular trajectories.

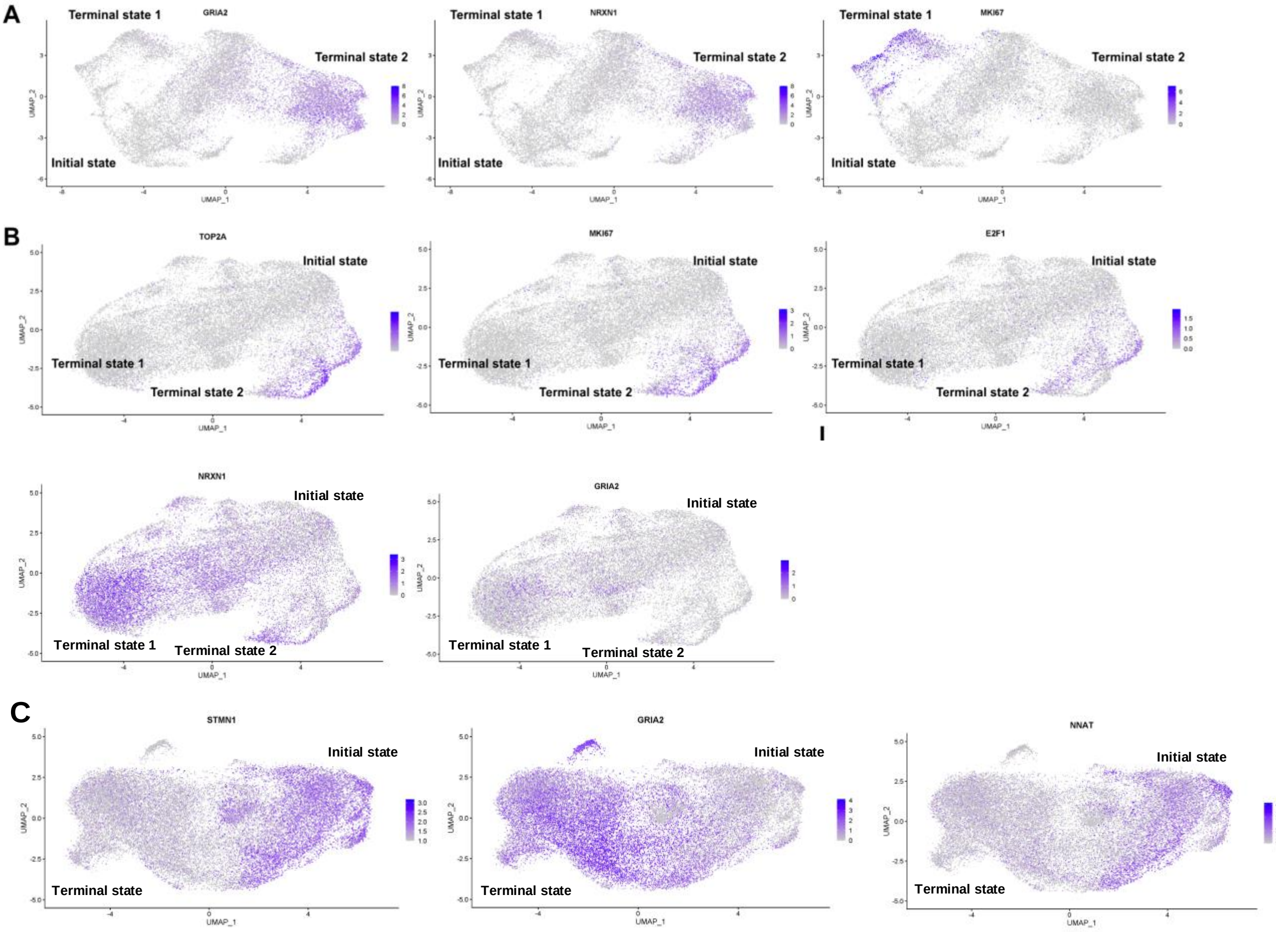

Suppl. Fig 7. Gene expression patterns in clusters representing initial and terminal states in MB subtypes. RNA velocity analysis was performed on the integrated SHH (**A**), Group 3 (**B**), and Group 4 (**C**) MB datasets using scvelo and scanpy functions in Python. The dynamical model of the RNA velocity and the Cellrank function were then used to identify the initial and terminal states of the cell trajectories.

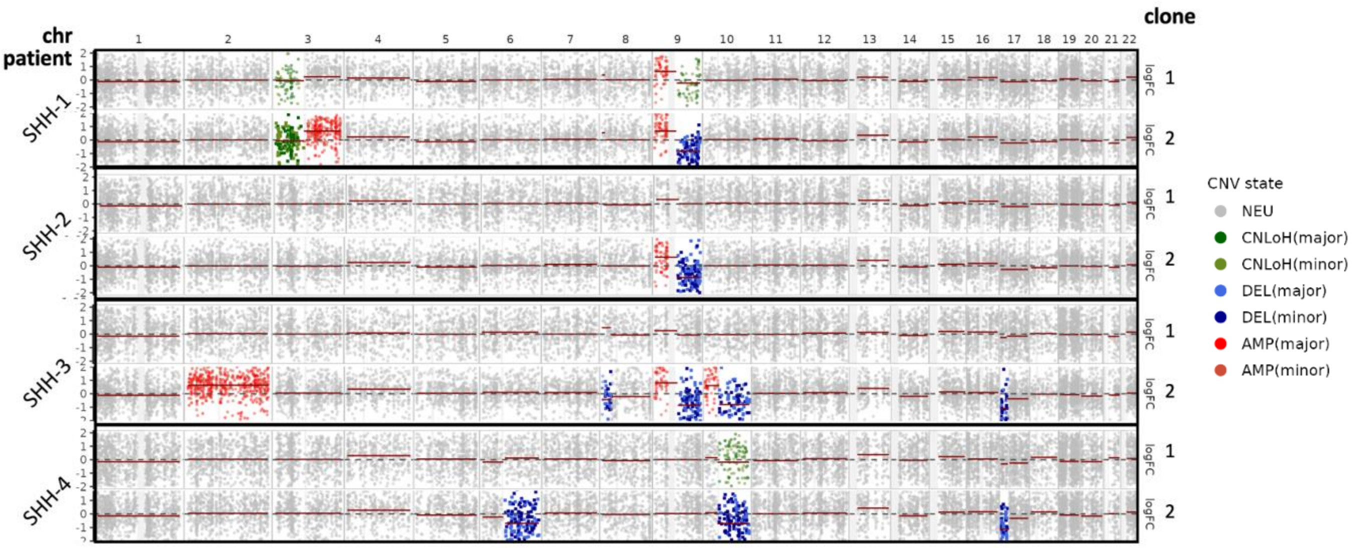

Suppl. Fig 8. Clone-specific CNVs in SHH MB. Four SHH tumour datasets sequenced using scRNA-seq were analysed using the Numbat pipeline (Gao et al., 2023). Immune and tumour cells from all 20 datasets combined were used as a reference. The following CNV states were identified: NEU = neutral, CNLoH = copy-neutral loss-of-heterozygosity, DEL = deletion, AMP = amplification.

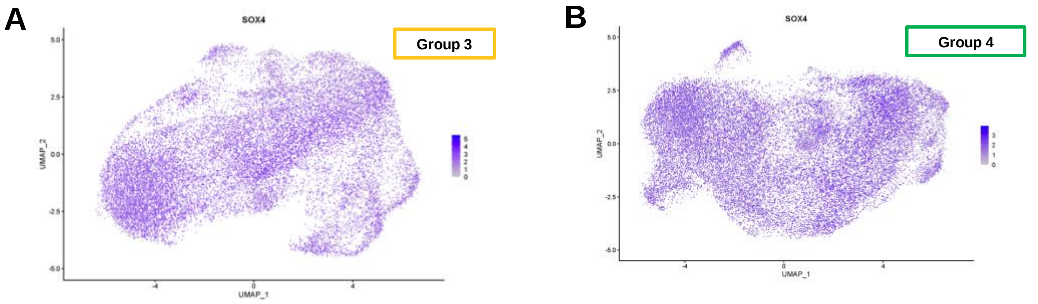

Suppl. Fig 9. Distribution of *SOX4* expression. Expression levels of *SOX4* gene on single-cell level were projected onto UMAPs of integrated Group 3 MB (**A**) and Group 4 MB (**B**) datasets.
