# Supplementary material for: Genomic tumor evolution dictates human medulloblastoma progression
Source: Neurooncol Adv. 2024 Oct 5;6(1):vdae172. doi: 10.1093/noajnl/vdae172 (PMC11629688; doi:10.1093/noajnl/vdae172)
Supplement: vdae172_suppl_Supplementary_Table_S1 [file vdae172_suppl_supplementary_table_s1.docx]

|  |  | |  | **Brain tumour classifier results** | | | | | | |
| --- | --- | --- | --- | --- | --- | --- | --- | --- | --- | --- |
|  |  | |  | **Methylation classes (MCs) with score >= 0.3** | | | **MC family members with score >= 0.1** | | | |
| **Number** | **Patient** | **Sex** | **Age**  (years:  months) | MC family MB, SHH | MC family MB, Wnt | MC family MB, group 3 and 4 | MC MB subclass SHH A (children and adult) | MC MB subclass SHH B (infant) | MC MB subclass group 3 | MC MB subclass group 4 |
| 1. | SHH-1 | F | 3:10 | 1.00 (match) |  |  | 0.89 (match) | 0.10 |  |  |
| 2. | SHH-2 | F | 0:9 | 1.00 (match) |  |  |  | 0.99 (match) |  |  |
| 3. | SHH-3 | F | 7:7 | 0.96 (match) |  |  | 0.61 (match) | 0.34 |  |  |
| 4. | SHH-4 | F | 3:5 | 1.00 (match) |  |  |  | 0.99 (match) |  |  |
| 5. | Wnt-1 | M | 11:3 |  | 0.99 (match) |  |  |  |  |  |
| 6. | Wnt-2 | M | 11:6 |  | 0.96 (match) |  |  |  |  |  |
| 7. | Wnt-3 | M | 5:8 |  | 0.98 (match) |  |  |  |  |  |
| 8. | Gr3-1 | F | 9:5 |  |  | 1.00 (match) |  |  | 1.00 (match) |  |
| 9. | Gr3-2 | M | 2:11 |  |  | 1.00 (match) |  |  | 1.00 (match) |  |
| 10. | Gr4-1 | M | 7:5 |  |  | 0.90 (match) |  |  | 0.11 | 0.78 (match) |
| 11. | Gr4-2 | M | 8:8 |  |  | 1.00 (match) |  |  |  | 1.00 (match) |
| 12. | Gr4-3 | M | 4:10 |  |  | 1.00 (match) |  |  |  | 1.00 (match) |
| 13. | Gr4-4 | M | 11:4 |  |  | 1.00 (match) |  |  |  | 1.00 (match) |
| 14. | Gr4-5 | M | 4:7 |  |  | 1.00 (match) |  |  |  | 0.99 (match) |

**Supplementary Table 1. Subtype diagnoses of 14 medulloblastoma patients based on methylation profile.** 14 MB patients treated at Karolinska University Hospital were diagnosed with different MB subtypes based on the tumour methylation classes using Heidelberg pipeline. Class descriptions were as follows:  **Methylation class family Medulloblastoma, SHH**: The methylation class family "Medulloblastoma, SHH" comprises the methylation classes medulloblastoma, SHH subtype A (children and adult) and medulloblastoma, SHH subtype B (infant). **Methylation class medulloblastoma, WNT**: The methylation class "medulloblastoma, WNT" is comprised of tumors diagnosed as Medulloblastoma, genetically defined, WNT activated. **Methylation class family Medulloblastoma group 3 and 4:** The methylation class family "Medulloblastoma class 3 and 4" comprises the methylation classes medulloblastoma, class 3 and medulloblastoma, class 4. The methylation class "medulloblastoma, subclass group 4" is comprised of tumors with the diagnosis medulloblastoma, genetically defined, group 4.
